# Novel Drugs in Follicular Lymphoma

**DOI:** 10.4084/MJHID.2016.061

**Published:** 2016-11-01

**Authors:** Antonella Anastasia, Giuseppe Rossi

**Affiliations:** SC Ematologia e Dipartimento di Oncologia Clinica, A.O. Spedali Civili, 25123 Brescia, Italy

## Abstract

Follicular lymphoma(FL) is the most common indolent non-Hodgkin lymphoma and constitutes 15% to 30% of lymphoma diagnoses. The natural history of the disease is characterized by recurrent relapses and progressively shorter remissions with a median survival of 10yrs. The impossibility of achieving a definite cure, have prompted investigations into the possible role of more active and less toxic strategies with innovative therapeutic agents. Recently *Casulo et al.* demonstrated that approximately 20% of patients with FL relapse within two years after achieving remission with R-CHOP and have a poor prognosis. It is conceivable that this particularly chemoresistant population would benefit from specifically targeting the biologic and genetic factors that likely contribute to their poor prognosis. Evolving strategies for difficult to treat FL patients have recently considered immunomodulatory agents, new monoclonal antibodies as well as drugs targeting selective intracellular pathways. The importance of targeting the microenvironment together with the malignant FL cell has been particularly underscored. We review the most promising approaches, such as combining anti-CD20 antibodies with immunomodulatory drugs (Lenalidomide), mAbs directed against other surface antigens such as CD22 and CD23 (Epratuzumab, Lumiliximab), immunomodulatory antibodies such as PD-1, or inhibitors of key steps in the B-cell receptor pathway signaling such as PI3K inhibitors (Idelalisib, Duvelisib). Another highly attractive approach is the application of the bi-specific T-cell engaging (BiTE) antibody blinatumomab which targets both CD19 and CD3 antigens. Moreover, we highlight the potential of these therapies, taking into account their toxicity. Of course, we must wait for Phase III trials results to confirm the benefit of these new treatment strategies toward a new era of chemotherapy-free treatment for follicular lymphoma.

## Introduction

Follicular lymphoma(FL) is the most common indolent non-Hodgkin lymphoma and constitutes 15% to 30% of lymphoma diagnoses. Its median survival is approaching ten years. The natural history of the disease is characterized by recurrent relapses and progressively shorter remissions. The impossibility of achieving a definite cure using the currently available chemo-immunotherapy regimens, as well as with more intensive treatments, such as high-dose therapy plus stem cell transplantation, have prompted investigations into the possible role of innovative therapeutic agents with more activity and less adverse events. Avoiding the toxic effects of chemotherapy would also be desirable for a disease with a relatively indolent course, where quality of-life is of primary importance, particularly in the elderly population.^1^

In addition, there are subsets of FL patients with a more aggressive disease who would also benefit from alternative treatment strategies. Recently, the US National LymphoCare Study have published data which show that approximately 20% of patients with FL relapse within two years from achieving remission with R-CHOP and have a poor prognosis, independent of that predicted by the FL International Prognostic Index (FLIPI). Their 5-year overall survival (OS) was only 50% compared to 90% in patients who had a longer treatment response.^2^

It is conceivable that this particularly chemo-resistant population would benefit from specifically targeting the biologic and genetic factors that likely contribute to the poor prognosis of this group. Indeed, the biological characteristics of FL and, more importantly, of its microenvironment, significantly impact on prognosis and may also play a significant role in determining FL sensitivity to treatments. A gene expression signature of the non-malignant stromal cells has been reported; that was prognostically more important than gene signatures deriving from the neoplastic B-cells.^3^

More recently, Pastore et Al. found that mutations in seven genes (EZH2, ARID1A, MEF2B, EP300, FOXO1, CREBBP, and CARD11), coupled with clinical parameters of FLIPI score and Eastern Cooperative Oncology Group (ECOG) performance status, were able to identify subgroups of FL patients with a distinct worse prognosis. This clinicogenetic risk model was termed m7-FLIPI.^4^

With the expanding knowledge of the pathogenesis of B-cell malignancies, in the last few years, several new therapies acting through a variety of mechanisms have shown promising results. We will briefly review the evidence available on these new drugs, which include new monoclonal antibodies and immunoconjugates, the anti-angiogenic and immunomodulatory agent lenalidomide, inhibitors of B-cell receptor pathway enzymes, such as ibrutinib, idelalisib, duvelisib and TGR-1202, BCL2 inhibitors, checkpoint inhibitors and CAR-Tcells (Table 1).

## New Generation mAbs

### AntiCD20 mAbs

The search for a monoclonal antibody with a higher activity than rituximab has been particularly active in recent years.

*Ofatumumab* is a humanized, class I anti-CD20 agent with an increased complement dependent cytotoxicity compared with rituximab. It binds to a different CD20 epitope resulting in higher affinity and, theoretically, a higher activity in cases with low CD20 surface expression.^5^ In a phase 3 trial including 116 FL patients previously treated with rituximab or rituximab-containing chemotherapy, ofatumumab monotherapy was well tolerated, but it showed an overall response rate (ORR) of only 10% in the 86 patients who received the highest dose (1000 mg/8 weekly doses).^6^ However, in first-line, in a phase 2 trial of FL patients, ofatumumab, given at 1000mg per week for a month and subsequently 1000 mg every 2 months for 8 months, obtained an ORR of 86% (Complete response [CR] in 13%) with a 1-year PFS probability of 97% and a safety profile similar to rituximab.^7^ It has also been administered as part of combination treatment; 59 patients with advanced-stage, previously untreated FL received ofatumumab plus CHOP (cyclophosphamide, doxorubicin, vincristine, prednisone) and attained an ORR of 100%, with CR in 62% of patients.^8^

*Obinutuzumab (GA101*) is another humanized anti-CD20 agent. It is a class II agent, and therefore, it has a higher antibody-dependent cellular cytotoxicity and induces B-lymphocyte apoptosis more effectively than rituximab.^9^

In patients with relapsed/refractory (R/R) indolent non-Hodgkin lymphoma (iNHL), eight cycles of obinutuzumab were administered (days 1 and 8 of the first cycle and day 1 of each subsequent cycle).^10^ One arm received 1600 mg in the first cycle and 800 mg subsequently, while the other arm received 400 mg (flat dose), obtaining an ORR of 55% and 17% and a median PFS of 11.9 and 6 months, respectively. In a phase II study including R/R iNHL patients, an induction and maintenance courses of obinutuzumab (4 weekly doses of 1000 mg and then every 2 months for 2 years) were superior in terms of ORR (45% vs. 27%) but not PFS to induction and maintenance with rituximab at the standard 375 mg/m^2^.^11^

GADOLIN (NCT01059630) is a phase 3 study including 396 rituximab-refractory iNHL patients. This randomized trial compared bendamustine alone (120 mg/m^2^ on days 1 and 2 of every 28-day cycle for up to six cycles) with bendamustine (90mg/m^2^) combined with obinutuzumab (1000 mg on day 1 of every cycle and days 8 and 15 of the first cycle), followed by maintenance with obinutuzumab. Both arms obtained the same ORR (63% vs. 69%), but a longer PFS was seen with the combination regimen followed by maintenance in the 321 patients (81%) with FL (13.8 months vs. not reached, Hazard ratio 0.48 [0.34–0.68]), despite a similar OS at the time of first analysis (albeit with less than 2 years of follow-up).^12^ Obinutuzumab-bendamustine was slightly more toxic, particularly due to an increased frequency of neutropenia (grade≥3 in 33%) and infusion reactions (grade≥3 in 8%), although there were fewer episodes of pneumonia (3%) and less thrombocytopenia (grade≥3 in 10%) than in the bendamustine monotherapy arm.

To date, the final results of the GAUSS Study, in which obinutuzumab (GA101) was prospectively compared with rituximab in a randomized fashion in relapsed indolent lymphoma, have been published.^13^ Among patients with follicular lymphoma, ORR seemed higher for obinutuzumab than rituximab (44.6% *v* 33.3%; *P* .08). However, this difference did not translate into an improvement in progression-free survival. No new safety signals were observed for obinutuzumab, and the incidence of adverse events was balanced between arms, with the exception of infusion-related reactions and cough, which were higher in the obinutuzumab arm.

The use of obinutuzumab in first-line therapy is under investigation, recruitment has been completed for the GALLIUM study (NCT01332968), a phase 3 randomized trial, including 1400 patients with previously untreated iNHL, comparing obinutuzumab vs. rituximab with combination chemotherapy. The results are still unpublished, but the interim analysis seems positive.

### Other B-lineage antigen-directed antibodies

Although targeting CD22 and CD23 by antibodies such as epratuzumab or lumiliximab was less successful when applied to single agents,^14^ the combination of epratuzumab with rituximab induced significant response rates both in relapse and front-line therapy.^15^ A similar activity was found when the CD22 antibody inotuzumab was conjugated with ozogamicin and its combination with rituximab.^16^ Promising data were also achieved by combining the anti-CD 80 antibody Galiximab with rituximab in patients with untreated FL.^17^

However, most of the above mentioned agents did not show sufficient activity to be further developed beyond the stage of clinical phase II studies.

More recently an anti-CD79B antibody has been successfully fused with the microtubule-disrupting agent monomethyl auristatin E. The resulting antibody-drug conjugate polatuzumab vedotin has undergone phase 1 and preliminary phase 2 studies and is currently under further investigation.^18^

## Drugs Targeting Oncogenic Pathways

### PI3K Inhibition

PI3K are enzymes involved in cell cycle regulation, apoptosis, DNA repair, senescence, angiogenesis and cell metabolism. Through their activation, cell surface signals are transmitted into the cytoplasm and, by phosphorylating different downstream molecules, they activate pathways such as BTK, AKT, PKC, NF-kappa-B and JNK/SAPK, which ultimately result in cell survival and growth. Its specific targeting has a strong rationale, given the interplay between lymphoma and microenvironment in FL. Different PI3K isoforms can be found in different cell types.^19^

*Idelalisib* is the first orally bioavailable, active inhibitor specifically targeting the delta isoform of PI3K, an enzyme downstream from the B cell receptor, which eventually signals through AKT and mTOR.^20^ Idelalisib showed marked antitumor activity in patients with FL who had not had a response to rituximab and an alkylating agent or had had a relapse within six months after those therapies (double-refractory patients). In a single-arm, open-label, phase 2 study, 125 patients with indolent non-Hodgkin’s Lymphomas were enrolled, including 72 FL patients refractory both to chemotherapy and rituximab.^21^ They received idelalisib, 150 mg twice daily, until disease progression. The 57% response rate was much higher compared to historical rituximab^22^ results in the same setting of refractory patients, with 14% meeting the criteria for a complete response. The median time to response was 1.9 months, the median duration of response was 12.5 months, and the median progression-free survival was 11 months. The most common adverse events of grade 3 or higher were neutropenia, in 27% of the patients, elevations in aminotransferase levels in 13%, diarrhea in 13%, and pneumonia in 7%).^21^

The results of this study led to the approval of idelalisib by the Food and Drug Administration (FDA) and the European Medicines Agency (EMA) for the treatment of FL relapsed after two previous lines of treatment (in the case of the EMA approval, refractoriness to the two previous lines of therapy is required).

However, because of an excess of severe infections, including Cytomegalovirus reactivation and Pneumocystis jirovecii pneumonia, in the idelalisib arm of three randomized trials, the safety of idelalisib particularly in the first line setting, is currently under reevaluation. Moreover, the association of idelalisib with lenalidomide and rituximab caused prohibitive hepatic toxicity in a phase I–II trial which had to be interrupted,^23^ underscoring the importance of an accurate evaluation of the risk benefit ratio of any new or any combination of no cytostatic agents. Other PI3k inhibitors currently under investigation include TGR-1202, duvelisib, and copanlisib.

*TGR-1202* is, like idelalisib, a selective delta isoform inhibitor. However, unlike the later, it showed very few autoimmune- mediated side effects in a recent analysis of 152 highly pretreated patients with CLL and NHL receiving TGR-1202 in monotherapy or in combination with the new anti-CD20 ublituximab.^24^ In the subgroup of R/R iNHL, the ORR was 49% in monotherapy and 71% (CR in 24%) when administered in combination.

*Duvelisib* (IPI-145), a gamma and delta isoform inhibitor, showed an ORR of 65% in a phase 1 dose-escalation study in patients with iNHL,^25^ which successfully met the primary endpoint of the phase II DYNAMO study.

The phase II study enrolled 129 patients with iNHL, which included follicular lymphoma (n = 83), small lymphocytic leukemia (SLL; n = 28), and marginal zone lymphoma (MZL; n = 18). The ORRs in each of these groups, respectively, were 41%, 68%, and 33%. The majority of adverse events reported in the study were labeled as clinically manageable and reversible.

Despite achieving the primary endpoint of the study, the magnitude of benefit did not match Infinity’s expectations. Based on the results, the long-term outlook for the medication was adjusted, studies exploring duvelisib in combination with venetoclax were paused, and 21% of the organization’s workforce was laid off as part of a research and development restructuring.^26^

*Copanlisib,* which inhibits the alfa and the delta isoform of PI3k, is also under active investigation and results of ongoing trials with this intravenously compound are awaited. To date, it seems to interfere with glucose metabolism.

### Bruton tyrosine kinase pathway inhibitors

The BTK pathway represents a further very attractive target in B-cell malignancies. Ibrutinib is the first-in-class among specific BTK-inhibitors.^27^

In R/R FL, in an update of the initial dose-escalation trial, ibrutinib monotherapy administered once daily at the dose of 560 mg until progression or withdrawal, has shown an ORR of 63% (CR in 38%) and a median PFS of 24 months. Among refractory NHL of different histologic subtypes, only minor activity was shown in patients with FL in contrast with patients with mantle cell lymphoma or Waldenstrom Macroglobulinemia, where results were much more promising.^28^ The combination of ibrutinib with chemoimmunotherapy (ICT) as well as with other agents including the combination of lenalidomide and rituximab is currently being tested. The preliminary results of a phase I-Ib study of the combination of ibrutinib with bendamustine and rituximab do not seem to confer a significant advantage over BR alone, but the number of patients treated was small.^29^

Phase 3 studies, such as SELENE (NCT01974440), which randomizes patients with R/R FL or marginal zone lymphoma to ICT (BR or RCHOP) with or without ibrutinib, are also underway.

### Bcl 2 inhibitors

Interesting data also emerged from agents interfering with the BCL2 family of proteins.

BCL2 is an antiapoptotic protein, typically overexpressed in lymphoma by the presence of its characteristic translocation t(14;18), which involves the BCL2 gene. However, anti-BCL2 drugs are proving active in a wide variety of B-cell disorders.^30^

Promising response rates were initially reported from a phase 1 study with ABT-263 (navitoclax), which is, however, associated with thrombocytopenia.^31^ A better efficacy-toxicity profile is revealed by the BH3 mimetic ABT-199 (venetoclax), already approved by FDA for B-CLL, which, in a phase 1 study, showed an ORR of 48% as a single agent. Given the direct involvement of bcl-2 in the pathogenesis of FL, the results achieved may be considered less favourable than expected. However, owing to its good tolerability profile, the combination of venetoclax with chemoimmunotherapy or other active agents is actively investigated and may yield better results.^32^ A phase 1b/2 trial with venetoclax, obinutuzumab and polatuzumab vedotin (NCT02611323) in R/R FL is currently recruiting patients. Trials with obatoclax, another BCL2 inhibitor, are also ongoing (NCT00438178, NCT00427856), but no results are available.

### M-TOR inhibitors and histone-deacetylase inhibitors

Temsirolimus is a mammalian target of rapamycin (mTOR) inhibitor, approved for the treatment of MCL, but which has also shown activity in FL. In 39 patients with R/R FL, at a weekly dose of 25 mg (intravenously), temsirolimus achieved an ORR of 54% (CR in 24%) and 3-year PFS and OS probabilities of 26% and 72%, respectively. Grade ≥3 neutropenia and thrombocytopenia were seen in around 30% of patients with all other toxicities, grade ≥3, occurring in ≤6% of patients.^33^ Temsirolimus is currently undergoing phase 3 trials in association with bendamustine and rituximab by the German study group.

The histone deacetylase inhibitor vorinostat has been studied in a phase II study in relapsed indolent lymphomas, which included 39 patients with FL, and demonstrated an encouraging 49% overall response rate with a median progression-free survival of 20 months in this small cohort.^34^

Everolimus too showed promising results in the relapsed/refractory setting with an ORR of 84 and 60%.^35^

## Immunomodulatory Drugs (IMiD)

*Lenalidomide* is one of the most promising new agents in the treatment of FL. Its mechanism of action is not fully understood but probably includes a modulation of the lymphoma microenvironment and an enhanced anti-lymphoma immune response.^36^ After promising single-agent activity was seen particularly in patients with refractoriness to prior therapies (ORR 23%, the median time to antitumor response 3.6 months, median PFS 4.4 months).^37^

Lenalidomide was combined with rituximab in the so-called R-squared or R2 regimen. The combination showed a higher ORR (76% vs. 53%) and longer time to progression (2 vs. 1.1 years), with no increased toxicity than lenalidomide alone.^38^ In the front-line setting, two recent publications reported results of phase 2 trials with the R-squared scheme. In the first, ORR was 98% (CR in 87%) with a 3-year PFS probability of 78% in a sub- group of 50 FL patients (out of 110 iNHL patients).^39^ The second trial carried out by the CALGB revealed an ORR of 93% (CR in 72%) and a 2-year PFS probability of 89% in 65 FL patients of whom only two discontinued treatment due to progressive disease and four due to toxicity.^40^

The same regimen has been subsequently compared with standard chemoimmunotherapy in a randomized phase 3 study (RELEVANCE) in untreated patients. Both arms are followed by a maintenance phase. Accrual has been completed but results are still not available.^41^

### Anti PD-1

Follicular lymphoma is characterized by a significant interplay between malignant cells and their microenvironment.^42^

PD-1 is a T-cell receptor which, upon binding its ligand (PD-L1 or PD-L2, found on B-cells), blunts T-cell response. PD-1 expression is an escape mechanism for a variety of solid and hematologic malignancies.^43^

The observation that some FL patients might have a very long survival without progressive disease after diagnosis and do not need anti-lymphoma therapy for years underscores the potential role of the activity of the immune system against the lymphoma cells. Although FL CD20+ tumor cells do not directly express PD1 ligands, PD-L1+ histiocytes are present within the T-cell-rich zone of the neoplastic follicles and contribute to the exhaustion of tumor PD1+ infiltrating T cells. Therefore, this subtype of lymphoma may represent a candidate for inhibiting the PD1–PD1-ligand axis.^44^

In a phase 1, open-label, single agent dose-escalation study, *Nivolumab*, an anti-PD1–blocking IgG4 antibody with higher affinity, yielded a response rate of 40% in 10 patients with relapsed/refractory FL, with a complete response rate of 10%, lending support to the importance of immune surveillance in the control and progression of this disease.^45^

*Pidilizumab* is an IgG1 isotype anti-PD1 antibody with lower affinity than other anti-PD-1 immune-checkpoint inhibitors. In 2014*,* the first results of a single group, phase 2 trial of pidilizumab combined with rituximab in relapsing follicular lymphoma were published. This combination showed a high objective response (66%) and complete response (52%,) with a median progression-free survival of 18 · 8 months (95% CI 14 · 7–not reached), an improvement on the expected results of rituximab alone in the same setting. No adverse events of grade 3–4 were reported.^46^

Although the actual benefit of this combination has to be established in a randomised study, these findings suggest that pidilizumab might enhance antitumor immune responses. The investigators correlated baseline gene expression signatures in tumor biopsy samples of 18 patients with the outcome of anti-PD1 therapy. They showed that a prominent T-cell activation signature or a signature of genes repressed in regulatory T cells were significantly associated with prolonged progression-free survival. Of major interest was the finding that a drug specifically targeting the immune system (pidilizumab) could be combined with drugs directly targeting the pathological cells (rituximab) to improve results.

To date, several trials with other checkpoint inhibitors such as ipilimumab, an anti-CTLA-4 (NCT01592370), and pembrolizumab, another PD-1 inhibitor, (NCT02446457), are ongoing.

## Bispecific T-cell Engagers and CAR-T Cells

Combining the specific targeting of the FL neoplastic cells obtained by mAb with the recruitment and activation of immune cells may ultimately be the most efficient strategy to achieve the definitive cure of FL.

A highly attractive approach is the application of the bi-specific T-cell engaging (BiTE) antibody blinatumomab which targets both CD19 and CD3 antigens. Although the development of this BiTE antibody is currently focused on B-precursor acute lymphoblastic leukemia, promising, early results were also obtained in B-cell lymphomas, where single agent blinatumomab at the dose of 60 ug/m^2^/day achieved an ORR of 80% in heavily pretreated FL patients, although with significant neurotoxicity with encephalopathy.^47^ Expanding clinical experience with the use of these molecule has reduced the important side effects initially observed. Moreover, subcutaneous formulations of blinatumumab have been developed and are currently being tested to allow its easier use in patients with lymphoma.

*Chimeric antigen receptor (CAR)-modified T cells* have generated broad interest in oncology following a series of dramatic clinical successes in patients with chemorefractory B cell malignancies. CAR therapy now appears to be on the cusp of regulatory approval as a cell-based immunotherapy.

Following a decade of preclinical optimization, CD19 chimeric antigen receptor (CAR) therapy has rapidly made a high impact in oncology. Within a few years, the CAR field has progressed from reports of anecdotal responses in patients with non-Hodgkin lymphoma (NHL) or chronic lymphocytic leukemia (CLL) to achieving reproducible outcomes in hundreds of patients with B cell malignancies, most strikingly in B cell acute lymphoblastic leukemia (B-ALL), including patients with chemotherapy-refractory disease.^48^ Indeed, the antitumor activity of anti-CD19 CAR was first reported in a case of advanced follicular NHL, in which anti-CD19 CAR therapy resulted in dramatic regression. Peripheral blood B-cells were absent for at least 39 weeks after treatment, but no acute toxicities occurred.^49^

This field is rapidly expanding both in the molecular engineering of new and more active products and in their clinical application in different hematological malignancies, including cases of FL.^50^ Nevertheless, the reproducibility and feasibility of CAR-T cell therapy on a scale broad enough to be offered to a large population of patients with CD19-positive malignancies need to be adequately demonstrated in more extensive studies.

The results of autologous CD19-targeted second-generation CAR T cells treatment obtained so far in indolent NHL patients have been recently updated.^51^ Of 15 patients enrolled, eight achieved complete remissions (CRs), four achieved partial remissions, one had stable lymphoma, and two were not evaluable for response. CRs were obtained by four of seven evaluable patients with chemotherapy-refractory DLBCL; three of these four CRs are ongoing, with durations ranging from 9 to 22 months. Acute toxicities including fever, hypotension, delirium, and other neurologic toxicities occurred in some patients after infusion of anti-CD19 CAR T cells; these toxicities resolved within three weeks after cell infusion. One patient died suddenly as a result of an unknown cause 16 days after cell infusion. The most troublesome toxicities experienced by patients were hypotension and neurologic toxicities. The mechanism of these neurologic toxicities is not entirely known. Elevated levels of interferon gamma and of interleukin-6 but not of tumor necrosis factor-alfa were common. Importantly, all patients recovered completely from their neurologic toxicities. Some of the neurologic toxicities observed were similar to toxicities observed in other trials of anti-CD19 CAR T cells and clinical trials of anti-CD19–and anti-CD3–bispecific antibodies.

In conclusion, in the last few years, several targeted drugs have shown activity in FL. Some of them like lenalidomide, selected PI3k inhibitors, and several new anti CD-20 monoclonal antibodies are likely to enter the clinical arena soon. Other compounds are still under clinical evaluation, like BTK-inhibitors, anti-bcl-2, and immunotoxins, whereas highly promising approaches, such as CART-cells and bispecific antibodies, need additional investigations to define their activity/toxicity and their best place in the treatment strategy for FL. All together they will certainly further expand our therapeutic armamentarium for FL patients including the highly refractory population that still has a dismal prognosis and no therapeutic options.

However, we must highlight that “chemo-free” treatments are not “toxicity-free,” which means that despite a different safety profile from that of the standard immunochemotherapy regimens, these new therapies continue to present significant and still incompletely known toxicities. The major trials, especially in the front-line setting and with combinations of different agents, have a limited follow-up period and involve relatively small patient populations. Thus, caution must be exercised in the interpretation of their results and only time will tell if they will ultimately replace our standard therapeutic approaches.

To conclude, we believe that the real challenge in the near future will be how to optimize and integrate in the clinical practice the results of the many studies reported, with the goal of achieving the cure of FL without compromising the quality of life of our patients. A further challenge, whose discussion is beyond the scope of the present review, will be how to optimize treatments also from an economic perspective. Given the high costs of all the novel agents described, the sustainability of every treatment program based on their use is likely to become equally important as their efficacy.

## Figures and Tables

**Table 1:** 

Drug category	Drug name	Target	Mecanism of action	Stage of clinical developement	Reference numbers

**Anti CD20**	Ofatumumab	CD20	ADCC/CDC	Phase 2–3	7,8
	Obinutuzumab			Phase 2–3	11,12,13

**Other cell surface-directed mAb**	Epratuzumab	CD22	ADCC/CDC	Phase 1	14–15
	Lumiliximab	CD23		Phase 1	14
	Inotuzumab			Phase 1	16
	Galiximab	CD80		Phase 1	17
	Polatuzumab	CD79b		Phase 1	18
	Blinatumumab	CD19/CD3	Engages CD3 T-cell killing of CD19 B-cell tumor cells	Phase 1	47

**Drugs targeting oncogeneic pathways**	Idelalisib	PI3Kδ	BCR pathway inhibition	Phase 2	22
	Duvelisib	PI3Kγ-δ		Phase 2–3	26
	TGR1202	PI3Kδ		Phase 1	24
	Copanlisib	PI3Kα-δ		Phase 2	

	Ibrutinib	BTK	BCR pathway inhibition	Phase 1/1b	29

	ABT263	BCL2	Reversing inhibition of apoptosis	Phase 1	31
	ABT199			Phase 1	32

	Vorinostat	mTOR	Histone deacetylase inhibition	Phase 2	34
	Temsirolimus			Phase 2–3	33
	Everolimus			Phase 1,3	35

**Immunomodulatory drugs (IMiD)**	Lenalidomide		Modulation of the lymphoma microenvironment Enhanced anti-lymphoma immune response	Phase 2–3	38–41

	Nivolumab	PD1	Inhibition of T-cell response blunting	Phase 1	45
	Pidilizumab	PD1		Phase 2	46
	Epratuzumab	PDL1		Phase 1	

**Chimeric antigen receptor (CAR)-modified T cells**	CAR-T cell	Anti-CD19	Chimeric antigen receptor (CAR)-modified T cells	Phase 1	49–51
